# Multiple imputation by predictive mean matching in cluster-randomized trials

**DOI:** 10.1186/s12874-020-00948-6

**Published:** 2020-03-30

**Authors:** Brittney E. Bailey, Rebecca Andridge, Abigail B. Shoben

**Affiliations:** 1 Department of Mathematics and Statistics, Amherst College, PO Box 5000, AC #2239, Amherst, 01002 MA USA; 2 College of Public Health, The Ohio State University, Columbus, 43210 OH USA

**Keywords:** Missing data, Cluster-randomized trial, Predictive mean matching, Multiple imputation

## Abstract

**Background:**

Random effects regression imputation has been recommended for multiple imputation (MI) in cluster randomized trials (CRTs) because it is congenial to analyses that use random effects regression. This method relies heavily on model assumptions and may not be robust to misspecification of the imputation model. MI by predictive mean matching (PMM) is a semiparametric alternative, but current software for multilevel data relies on imputation models that ignore clustering or use fixed effects for clusters. When used directly for imputation, these two models result in underestimation (ignoring clustering) or overestimation (fixed effects for clusters) of variance estimates.

**Methods:**

We develop MI procedures based on PMM that leverage these opposing estimated biases in the variance estimates in one of three ways: weighting the distance metric (PMM-dist), weighting the average of the final imputed values from two PMM procedures (PMM-avg), or performing a weighted draw from the final imputed values from the two PMM procedures (PMM-draw). We use Monte-Carlo simulations to evaluate our newly proposed methods relative to established MI procedures, focusing on estimation of treatment group means and their variances after MI.

**Results:**

The proposed PMM procedures reduce the bias in the MI variance estimator relative to established methods when the imputation model is correctly specified, and are generally more robust to model misspecification than even the random effects imputation methods.

**Conclusions:**

The PMM-draw procedure in particular is a promising method for multiply imputing missing data from CRTs that can be readily implemented in existing statistical software.

## Background

A cluster-randomized trial (CRT) is a trial in which intact groups, or *clusters*, rather than individuals are randomized to treatment conditions. As with any study, CRTs are plagued by problems with dropout and nonresponse. The added statistical challenge for CRTs is that the assumption of independence is violated: subjects within the same cluster tend to have correlated outcomes. When data are missing in a CRT, procedures used to fill in, or *impute*, the missing data should account for this correlation; failure to do so may result in inflated Type I error rates [1] and thus misleading conclusions.

Fully parametric procedures for imputing missing data from a CRT are commonly used in practice, but a semiparametric procedure for imputation should be more robust to misspecification of the imputation model. In this article, we propose three new semiparametric procedures for multiple imputation and compare them to established methods for CRTs.

### Estimation and inference after multiple imputation

When data are missing from a CRT, how we handle the missing data depends in part on the reason behind the missing data. These missing data mechanisms are commonly classified into one of three types: missing completely at random, missing at random, or missing not at random [2]. We say data are *missing completely at random* (MCAR) if the probability that data are missing does not depend on any observed or unobserved data. A more reasonable assumption in many cases is that the data are *missing at random* (MAR), meaning the probability that data are missing depends on the observed data, but does not depend on any missing observations. Lastly, we say data are *missing not at random* (MNAR) if the probability that data are missing depends at least in part on unobserved data (e.g., the observations that are missing).

Multiple imputation (MI) is a common procedure for handling missing data that is valid under MAR and involves three phases: imputation, analysis, and pooling. In the *imputation phase*, we copy the incompletely observed data *D* times and augment each incomplete dataset with different imputed estimates of the missing values. Then in the *analysis phase*, we analyze each of the *D* imputed datasets using the planned method of analysis for the study (e.g., random effects models for CRTs). Finally, in the *pooling phase*, we use Rubin’s rules to combine the results of the *D* analyses to produce a single result [3]. We are primarily concerned with the imputation phase in this paper, but for completeness we describe the analysis and pooling phases below.

Assume we measure a continuous outcome *y*_*ijl*_ and a set of covariates *x*_*ijl*_ for subject *i*=1,…,*m* in cluster *j*=1,…,*k* of study condition *l*=1,2 for a total of 2*km* participants. The data generating model is *y*_*ijl*_=*xijl*′*β*+*b*_*jl*_+*e*_*ijl*_ where the cluster-specific intercepts $b_{jl} \overset {\text {i.i.d.}}{\sim } N\left (0,\,\sigma ^{2}_{b}\right)$ and are mutually independent of the random error terms, $e_{ijl} \overset {\text {i.i.d.}}{\sim } N\left (0,\,\sigma ^{2}_{e}\right)$. The total variation in *y*_*ijl*_ is $\sigma ^{2}=\sigma ^{2}_{b} + \sigma ^{2}_{e}$, and the intracluster correlation coefficient (ICC), which measures the within-cluster association, is given by $\rho = \sigma ^{2}_{b}/\sigma ^{2}$.

Of primary interest is the *l*th treatment group mean, *θ*_*l*_, estimated in complete data by $\hat {\theta }_{l} = \bar {y}_{\cdot \cdot l}$ with corresponding variance

$$  \text{Var} \left({\hat{\theta}_{l}}\right) = \frac{\sigma^{2}}{km}\left[ 1 +(m-1)\rho \right]. $$

When *σ*^2^ and *ρ* are unknown, we can estimate this quantity with

$$ \widehat{\text{Var}} \left({\hat{\theta}_{l}}\right) = \frac{1}{2k(k-1)} \sum_{l=1}^{2}\sum_{j=1}^{k}(\bar y_{\cdot jl} - \bar y_{\cdot \cdot l})^{2}, $$

using the ANOVA method [4]. If we only observe outcomes for the first *r* out of *m* subjects (without loss of generality) in each of the *k* clusters and we impute the remaining *m*−*r* values with $y_{ijl}^{(d)}$, then the estimator of the treatment group mean in the *d*th imputed dataset is

$$ \hat {\theta}_{l}^{(d)} = \frac{1}{km} \left(\sum_{j=1}^{k} \sum_{i=1}^{r} y_{ijl} + \sum_{j=1}^{k} \sum_{i=r+1}^{m} y_{ijl}^{(d)}\right) $$

with estimated variance

$$ W_{l}^{(d)} =\widehat{\text{Var}} \left(\hat {\theta}_{l}^{(d)} \right) = \frac{1}{2k(k-1)} \sum_{l=1}^{2}\sum_{j=1}^{k} \left(\bar y^{(d)}_{\cdot jl}- \bar y^{(d)}_{\cdot \cdot l}\right)^{2}. $$

Pooling these results for *d*=1,…,*D*, the MI estimator of *θ*_*l*_ is simply $ \hat {\theta }^{\text {MI}}_{l} = \frac {1}{D}\sum \hat {\theta }_{l}^{(d)} $. The corresponding variance estimator after MI captures both the variability within each imputed dataset $\left (\overline {W}_{l} = \frac {1}{D}\sum W_{l}^{(d)}\right)$ and the variability between the *D* imputed datasets $\left (B_{l} = \frac {1}{D-1}\sum \left [\hat {\theta }_{l}^{(d)}-\hat {\theta }_{l}^{\text {MI}} \right ]^{2}\right)$. The MI variance estimator is

$$  \widehat{V}_{l}^{\text{MI}} = \widehat{\text{Var}}\left(\hat \theta_{l}^{\text{MI}}\right) = \overline{W}_{l} + \left(1+\frac{1}{D}\right)B_{l}, $$

where the factor $\left (1+\frac {1}{D}\right)$ corrects the bias due to the finite number of imputed datasets [3]. Inference on *θ* can proceed by assuming the test statistic

$$  t_{\nu}=\frac{ \hat{\theta}_{l}^{\text{MI}} - \theta_{l} }{ \sqrt{ \widehat{V}_{l}^{\text{MI}}} } $$

follows a *t* distribution with *ν* degrees of freedom. The typical formula for the degrees of freedom after MI is

$$  \nu = (D-1)\left(1+ \frac{D}{D+1}\frac{\overline{W}_{l}}{B_{l}} \right), $$

but a different formula should be used in small samples or in study designs with limited degrees of freedom, as in CRTs where the degrees of freedom are driven by the number of clusters rather than the number of subjects [2, 5]. In this case, the degrees of freedom are $ \nu ^{*} = \left (\nu ^{-1} + \hat {\nu }_{\text {obs}}^{-1} \right)^{-1} $, where $\hat {\nu }_{\text {obs}} = {\left (\frac {\overline {W}_{l}}{\widehat {V}_{l}^{\text {MI}}} \right)}{ \left (\frac {\nu _{\text {com}}+1}{\nu _{\text {com}}+3} \right)}$ and *ν*_com_ are the degrees of freedom based on complete data.

### Fully parametric imputation

While estimation and inference for CRTs are fairly straightforward after multiple imputation, the imputation phase itself poses the greatest challenge. Ideally, the imputation model is congenial to the analysis model, meaning the model used to analyze the data can be derived from the imputation model [6]. For CRTs, this means we should use a random effects model to impute the missing data. Some authors have shown this method to work well in limited simulations [1, 7], but it relies heavily on model assumptions. Additionally, the number of clusters may be quite small for some CRTs, and random effects models may not be the most appropriate or most powerful analysis approach [8, 9].

Although the random effects model is an appropriate congenial imputation model for CRTs, models that ignore clustering or model clusters as fixed effects have been used in practice [10–13]. For continuous outcomes, the imputation model that ignores clustering is the standard regression model:

$$  \begin{aligned} y_{ijl} &= \pmb{x}_{ijl}'\pmb{\beta} + e_{ijl},\\ e_{ijl} & \overset{\text{i.i.d.}}{\sim} N(0,\,\phi^{2}_{e}). \end{aligned} $$

We use $\phi ^{2}_{e}$ here to distinguish the imputation model parameters from the data-generating parameters of the random effects model. The fixed effects imputation model is similar, with the intercept term replaced by the following set of indicators:

$$\sum_{g=1}^{2}\sum_{c=1}^{k}\beta_{0cg}\pmb{1}(c=j,\,g=l).$$

Neither of these models are congenial to a random effects analysis model, but they still produce unbiased estimates of the treatment group mean. However, these imputation models result in biased estimates of the variance when used for fully parametric multiple imputation [1, 7]. Specifically, assuming the data are MCAR and the response rate *π* is the same for all clusters, the bias of the multiple imputation variance estimator based on the fixed effects (FE) imputation model as *D*→*∞* is

1 $$  \text{Bias}_{\texttt{FE}}\left(\hat V^{\text{MI}}_{l} \right) = \frac{2(1-\pi)(1-\rho)\sigma^{2}}{km\pi},  $$

which is always positive [7]. This overestimation is worse with smaller ICCs and smaller cluster sizes. On the other hand, the bias of the MI variance estimator when ignoring clustering (IGN) in the imputation model is

2 $$  \begin{aligned} \text{Bias}_{\texttt{IGN}}\left(\hat V^{\text{MI}}_{l} \right) = \frac{\sigma^{2}\rho}{km\pi-1}(m\pi-2)(\pi^{2}-1), \end{aligned}  $$

which is always negative and worsens with larger ICCs. The derivation of (2) can be found in Additional File 1.

### Imputation by predictive mean matching

An alternative approach to imputation that is relatively less reliant on model assumptions is predictive mean matching (PMM), where the missing outcome for a nonrespondent (or *recipient*) is imputed by the observed outcome from a respondent (or *donor*) with a similar predicted mean outcome [14]. We outline a typical PMM procedure here for recipient 0:

1. For a partially observed outcome *y*, use regression models to obtain predicted means ($\hat y_{i}$) for all subjects and a posterior predicted mean ($y^{*}_{0}$) for recipient 0.
2. Find a pool of *K* donors that minimize the distance $d(0,\, i) = |{y}^{*}_{0} - \hat {y}_{i}|$.
3. Randomly select a single donor from the donor pool, and use the observed value from this donor as the imputed value for recipient 0.

To multiply impute, we repeat this procedure *D* times and proceed with the analysis and pooling phases as described previously. There are many different variants of PMM, based on varying the way the predicted means are calculated, the size of the donor pool, and the way a donor is selected from the pool (e.g., [15–17]). For a comprehensive review of the many flavors of PMM, see [14].

Multiple imputation using predictive mean matching has many desirable properties, particularly when data do not satisfy the assumption of multivariate normality that is common in fully parametric imputation methods. PMM may be more robust to model misspecification than fully parametric imputation methods since parametric assumptions are only present in the metric used to match donors to recipients. In addition, only plausible values are imputed for the missing data, allowing distributions to be preserved. In practice, PMM is also computationally simpler than fully parametric imputation methods. We are therefore interested in extending the PMM algorithm to multiple imputation of CRT data in a manner that is both convenient and statistically valid.

While PMM has been shown to work well in single-level data, it is not clear how best to extend the procedure to multilevel data as found in CRTs. To emulate fully parametric imputation, a natural extension is to include random effects for clusters in the predictive mean models. This would involve using the best linear unbiased prediction estimates to obtain $\hat {y}_{ijl} = \pmb {x}_{ijl}'\pmb {\beta } + \hat {b}_{0jl}$, and implementing a Gibbs sampler to obtain posterior draws of *β* and *b* for the posterior predictive mean model [18]. Although this approach is a natural way to extend the PMM matching procedure to multilevel data and is recently available in an R package [19], it has not been extensively evaluated.

To our knowledge, only one group of authors has recommended a PMM procedure for multiple imputation of multilevel data. Vink et al. in 2015 [20] proposed incorporating cluster indicators as fixed effects in the predictive mean model to account for between-cluster variability. They used simulation to compare a PMM procedure that included fixed effects for clusters to two fully parametric MI procedures that used random effects for clusters, evaluating bias in the estimated cluster means, coverage rate of the corresponding 95% confidence intervals, and the estimated ICC. The authors found that the PMM procedure with a fixed effects model performed as well as the fully parametric procedures across all cases, and that the PMM procedure actually outperformed the parametric procedures when there was a large amount of missing data or when cluster-level variables were missing.

Despite these promising results, the simulation in [20] involved a single data set, and we suspect that the results are not fully generalizable to a range of ICCs and cluster sizes. Thus, more work is needed to evaluate the use of PMM for multilevel data arising from CRTs.

In this paper, we use our knowledge of the biases described in Eqs. 1 and (2) to propose new PMM procedures for multiple imputation of multilevel data that might mitigate the bias in the MI variance after MI and can be implemented using current software. We evaluate these methods in an extensive simulation study, comparing them to both fully parametric imputation methods as well as PMM methods based on fixed effects for clusters, ignoring clusters, and the random effects model. We also apply the new methods to data from the Work, Family, and Health Study [21].

## Methods

We know that ignoring clustering in the imputation model results in *underestimation* of the variance of the treatment group mean, while fixed effects imputation results in *overestimation* of the variance, so neither of these approaches alone are appropriate for multiple imputation of CRT data. While this bias is a direct result of the imputation model for fully parametric procedures, the imputation model only controls the donor pool for predictive mean matching procedures. To reduce this bias without changing the imputation model itself, we need to adjust the donor pool so that the final imputed values, and thus the pooled estimates themselves, better reflect the true variability after multiple imputation. We propose to do this by allowing both models to contribute to the donor pool in an amount inversely proportional to the magnitude of its expected bias. In the extreme, if one model is expected to produce an unbiased estimate of the MI variance, it will be the only model contributing to the donor pool. Capitalizing on the opposing directions in bias, we propose three new weighted PMM procedures that combine both an imputation model that uses fixed effects for clusters and one that ignores clustering.

***Choice of weights***

We require weights that sum to one and that favor the imputation model with the smaller magnitude of the bias. We propose the following weight for the predictive mean model that ignores clustering, noting that *w*_FE_=1−*w*_IGN_:

$$  w_{\texttt{IGN}} = \frac{|\text{Bias}_{\texttt{FE}}|} {|\text{Bias}_{\texttt{IGN}}|+|\text{Bias}_{\texttt{FE}}|} $$

Plugging in the derived formulas for the biases in (1) and (2) and assuming $\frac {\sigma ^{2}}{km\pi }\approx \frac {\sigma ^{2}}{km\pi -1}$, we get

$$ w_{\texttt{IGN}} = \frac{|2(1-\pi)(1-\rho)|} {|\rho(m\pi-2)(\pi^{2}-2)| + |2(1-\pi)(1-\rho)|}. $$

To estimate these weights, we use the observed response rate for *π* and estimate *ρ* from a complete case analysis of the data. Alternatively, depending on the extent and nature of the missing data, we might consider using a prior estimate of the ICC from similar studies. We note that the bias formulae were derived assuming a balanced design with equal numbers of respondents and nonrespondents in each cluster. It is unlikely that the number of respondents per cluster is the same across all clusters, so in practice *mπ* in the formula can be replaced by $\bar {r}$, the average number of respondents per cluster.

***PMM-dist: minimize the weighted distance***

The PMM imputation procedure can be modified at one of two stages to incorporate the weighting of the two single-level imputation models: the creation of the donor pool (e.g., via definition of the distance metric) or the final donor selection. Our first proposed procedure, which we call PMM-dist, modifies the definition of the distance metric (and therefore creation of the donor pool). We define a new distance metric, *d*^∗^(0,*i*), that weights the magnitude of the distances from each single-level imputation procedure. We calculate the distances *d*_IGN_(0, *i*) and *d*_FE_(0, *i*) under each predictive mean model and combine them in a final distance metric:

$$  d^{*}(0,\,i)= w_{\texttt{IGN}}d_{\texttt{IGN}}(0,\,i)+ w_{\texttt{FE}}d_{\texttt{FE}}(0,\,i). $$

The PMM procedure then proceeds as usual: we find the *K* donors that minimize *d*^∗^(0,*i*) and randomly select a single donor from this pool. Relative to the two methods we describe next, the downside of this method is that it cannot be implemented in existing software since creation of a new distance metric requires internal modification of the PMM algorithm.

***PMM-draw: randomly draw from final two donors***

To make implementation easier in available software, we can instead weight the final donor selection after producing a donor from each imputation procedure separately. That is, if we use PMM with a model that ignores clustering and select *donor*_IGN_, and use PMM with fixed effects for clusters and select *donor*_FE_, then we can select one of these two donors for final imputation with corresponding probabilities *w*_IGN_ and *w*_FE_. This is easily implemented in existing software by running the two imputation procedures separately, for each recipient generating a random draw, *S*, from a Bernoulli distribution with probability *w*_IGN_, then selecting *donor*_IGN_ if *S*=1, and selecting *donor*_FE_ otherwise. This method, which we call PMM-draw, is equivalent to selecting a donor from a combined pool of 2*K* donors (*K* from each predictive mean model), where the weight for each donor is either $\frac {1}{K}w_{\texttt {IGN}}$ or $\frac {1}{K}w_{\texttt {FE}}$.

***PMM-avg: impute weighted average from final two donors***

Our third proposed procedure, which we call PMM-avg, combines the observed values of the final donors produced by each imputation procedure. Let *y*_IGN_ represent the observed value from *donor*_IGN_ and *y*_FE_ represent the observed value from *donor*_FE_. We define the final imputed value as the weighted average of these two values; that is, $\dot {y}=w_{\texttt {IGN}}y_{\texttt {IGN}} + w_{\texttt {FE}}y_{\texttt {FE}}$. As with the PMM-draw method, this procedure can be readily implemented in existing software. One potential disadvantage, however, is the possibility of imputing values that are not observed or plausible (for example, with discrete data).

### Simulation study

We conducted a two-part simulation study to evaluate the performance of the proposed PMM procedures (PMM-dist, PMM-draw, and PMM-avg) relative to six existing methods for multiple imputation of CRT data that are available in current software. The six existing methods were split between fully parametric procedures (NORM-IGN, NORM-FE, and NORM-RE) and PMM procedures (PMM-IGN, PMM-FE, and PMM-RE). As before, IGN indicates an imputation or predictive mean model that ignores clustering; FE indicates a model that uses fixed effects for clusters; and RE indicates a model that uses random effects for clusters.

We generated continuous outcomes for *k* clusters of equal size *m* for each of two study conditions using the three models below:

$${\begin{aligned} \text{ Model 1a:} y_{ijl} &=\beta_{0}+\beta_{1}\texttt{Trt}_{l}+b_{jl}+e_{ijl}\\ \text{ Model 1b:} y_{ijl} &=\beta_{0}+\beta_{1}\texttt{Trt}_{l}+ \beta_{2} x_{ijl} + b_{jl}+e_{ijl}\\ \text{ Model 2:} y_{ijl} &=\beta_{0} +\beta_{1}\texttt{Trt}_{l}+\beta_{2}x_{ijl}+\beta_{3}{x^{2}_{ijl}}+b_{jl}+e_{ijl} \end{aligned}} $$

where Trt_*l*_ was the treatment group indicator and *x*_*ijl*_ was a continuous covariate.

In the first part of the simulation, Models 1a and 1b were used to establish how well the proposed PMM procedures for CRTs performed in ideal circumstances for multiple imputation; that is, the data were MCAR, and imputation models for data generated from Model 1a correctly included only the treatment group indicator as a covariate effect while imputation procedures for Model 1b correctly included both the treatment group indicator and the continuous covariate. In the second part of the simulation, Model 2 was used to examine how robust each procedure was to misspecification of the mean structure in the imputation models. Here, imputation models incorporated only the treatment effect and a linear effect of *x*_*ijl*_, ignoring the squared term.

To simplify exposition and without loss of generality, we set *β*_0_=*β*_1_=0 for all models. We fixed the total variance at *σ*^2^=16 across simulations, with the cluster-specific intercepts generated as *b*_*jl*_∼*N*(0, *ρσ*^2^) and the residual errors generated as *e*_*ijl*_∼*N*(0, (1−*ρ*)*σ*^2^). The continuous covariate *x*_*ijl*_ in Models 1b and 2 was generated as *x*_*ijl*_∼*N*(1, 1) and remained fixed for all simulations. For Model 1b, we set *β*_2_=3. For Model 2, following [14], we set *β*_2_=0 and *β*_3_=3.33. This corresponds to a strong association between *x*_*ijl*_ and *y*_*ijl*_ (*R*^2^≈0.8).

We considered (*k*,*m*)=(4,400), (10, 8), (10, 40), (20, 8), (20, 40), (40, 8), (40, 40), (100, 4), which reflect typical study sizes from a variety of CRT designs, including community-based trials, small psychotherapy trials, site-randomized trials, and family-based trials. To reflect the typical range of ICCs in CRTs, we simulated data with *ρ*=0.01, 0.03, 0.08, 0.15.

We generated missing data completely at random (MCAR) with response rates of *π* = 0.60, 0.85. For Model 2 only, we additionally generated missing data at random (MAR) using a logistic regression model with the coefficient of the covariate *x*_*ijl*_ chosen such that subjects with smaller values of *x* were more likely to have observed outcomes. The strength of the association between *x*_*ijl*_ and *P*(*R*_*ijl*_=1) was defined as either weak (log odds ratio of −1.25) or strong (log odds ratio of −2.5). The intercept in the logistic regression model was chosen so as to produce the desired response rate. Because the imputation procedures under Model 1 correctly included all data-generating covariate effects, there was no need to additionally evaluate the imputation procedures under an MAR missing data mechanism.

Data were imputed *D*=50 times for all nine imputation procedures. For all PMM procedures, we produced pools of *K*=5 donors that were matched to recipients based on Type 1 matching, where predicted means for donors are based on the maximum likelihood estimates from the regression models, but predicted means for recipients are based on draws of their posterior predicted means [14]. After imputation, we estimated the treatment group means for Models 1a and 2, and only the regression coefficient *β*_2_ for Model 1b. We used Rubin’s Rules to pool the imputed estimates and then tested whether the MI estimate was different from zero using the *t* test with small sample degrees of freedom as described earlier.

We repeated this process 1000 times for each of the nine imputation procedures, and evaluated the performance of each procedure based on (1) bias in estimation of the treatment group mean or the coefficient of the continuous covariate, (2) magnitude of the empirical variance, (3) relative percent error in the pooled standard error after multiple imputation, $\sqrt {\hat V^{\text {MI}}}$ (or *MI standard error*), and (4) empirical coverage of 95% confidence intervals for the treatment group mean. Bias in estimation of the treatment group mean or the regression coefficient was calculated as the average of the 1000 mean estimates or coefficient estimates, denoted $\bar \theta $, minus the true data-generating treatment group mean or regression coefficient. The relative percent error in the pooled standard error (SE) was calculated as $100\left (\frac { \widehat {\text {SE}}^{\text {MI}} }{ \widehat {\text {empSE}}} - 1 \right)$, where $\widehat {\text {SE}}^{MI} = \sqrt { \frac {1}{1000} \sum _{I=1}^{1000} \hat V_{I}^{\text {MI}} }$ and $\widehat {\text {empSE}} = \sqrt { \frac {1}{1000-1} \sum _{I=1}^{1000} \left (\hat \theta _{I}^{\text {MI}} - \bar {\theta } \right)^{2} }$ [22]. Results are presented with their corresponding Monte Carlo 95% confidence intervals (see [22] for specific formulas).

## Results

### Simulation study

Results from the simulation study are described only for the data generated with a response rate of 60%. When the response rate was 85%, the direction of any observed biases was the same, but the magnitude of any biases was typically smaller. Where the magnitude of the ICC had an impact, only *ρ*=0.03 versus *ρ*=0.08 are shown for contrast. Increasing the size of the study generally improved estimation, with increases in cluster size showing a greater effect than increases in number of clusters. Results are therefore shown for only four of the eight sample sizes considered: (*k*,*m*) = (100,4), (20,8), (20,40), (4,400). Complete results are available from the authors upon request.

#### Model 1a: estimation of treatment group mean with correctly specified mean model

When data were MCAR and the models used for imputation included the appropriate data-generating covariate effects, estimation of the treatment group mean after multiple imputation was unbiased for all MI procedures, regardless of the response rate (not shown).

Although PMM methods yielded slightly smaller estimates of the empirical variance relative to parametric methods, there were no meaningful differences in efficiency across imputation methods with the exception of the PMM-RE approach, which was less efficient than all other methods in this setting (Supplemental Figure 1).

**Fig. 1:**
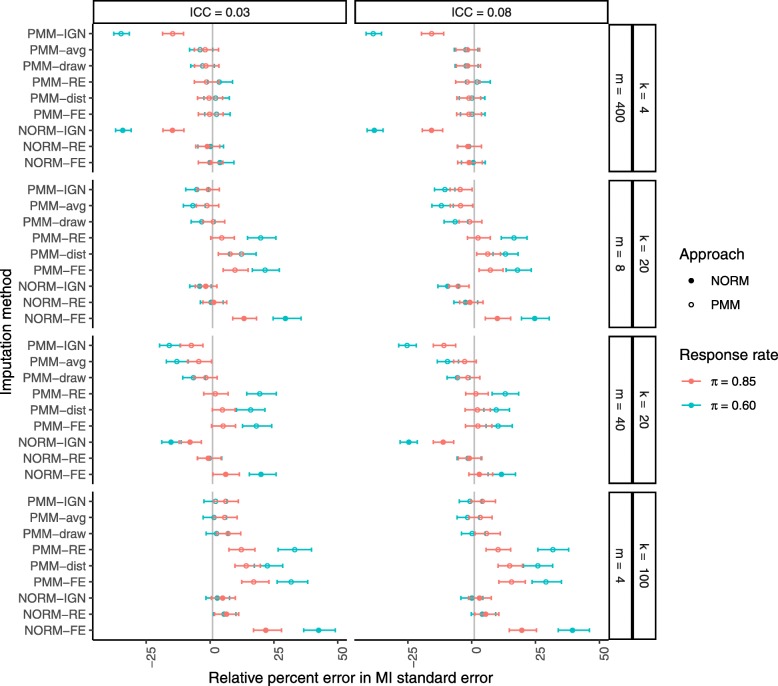
Relative percent error in the MI standard error of the treatment group mean after MI with a correctly specified imputation model. Missing data were generated completely at random (MCAR) at a rate of 15% or 40% (*π*=0.85 in red or *π*=0.60 in green, respectively). From top to bottom, results are presented in increasing order of cluster sizes *m* within an increasing order of number of clusters *k*. From left to right, results are presented in increasing order of the intracluster correlation coefficient (ICC, *ρ*). Filled circles indicate PMM methods while open circles indicate parametric (NORM) methods

Figure 1 shows the relative percent error in the MI standard error of the treatment group mean. As expected and as previously shown, ignoring clustering in the imputation procedure generally underestimated the empirical standard error, and the bias was worse for larger ICCs, larger cluster sizes, and higher rates of missing data. Modeling clusters as fixed effects, on the other hand, generally overestimated the empirical standard error, more so for smaller ICCs, smaller cluster sizes, and higher rates of missing data. We observed this same pattern of biases in the results from the PMM-RE and PMM-dist procedures, although they both offered some improvement over the PMM-FE and NORM-FE procedures. Among all methods considered, the newly proposed PMM-draw procedure was the only one to produce unbiased estimates of the empirical standard error in all scenarios. The PMM-avg procedure performed similarly, with some underestimation of the variance when there was a higher rate of missing data.

Coverage rates for 95% confidence intervals for the treatment group mean are shown in Tables 1 and 2 and correspond with the relative percent error in the MI standard error: methods where the MI standard error overestimated the empirical SE resulted in overcoverage of the 95% CIs, whereas methods where the empirical SE was underestimated resulted in undercoverage.

**Table 1:** Empirical coverage of 95% confidence intervals for the treatment group mean after multiple imputation by PMM

ICC	Cluster size	Clusters	PMM	PMM	PMM	PMM	PMM	PMM
*ρ*	*m*	*k*	FE	dist	RE	draw	avg	IGN
0.03	4	100	*99.0*	*98.2*	*98.7*	95.1	95.4	95.5
	8	10	*99.5*	*98.1*	*98.1*	*97.5*	*96.7*	*97.1*
		20	*98.4*	*96.6*	*96.8*	94.9	94.9	95.2
		40	*98.9*	*97.8*	*97.5*	95.6	95.3	95.4
	40	10	*98.9*	*98.4*	*98.9*	96.1	94.9	94.4
		20	*98.3*	*98.0*	*98.1*	94.5	**92.3**	**91.7**
		40	*98.0*	*98.0*	*98.2*	94.0	**92.1**	**91.0**
	400	4	*97.5*	*97.3*	*98.1*	96.2	95.5	**89.9**
0.08	4	100	*98.7*	*98.6*	*98.2*	94.9	94.8	95.0
	8	10	*98.9*	*97.5*	*98.0*	*96.7*	95.0	96.0
		20	*97.9*	*97.1*	*96.9*	93.9	**92.3**	**93.6**
		40	*98.9*	*98.7*	*98.2*	95.0	93.7	94.2
	40	10	*97.9*	*97.7*	*98.7*	95.3	93.9	**90.2**
		20	*97.6*	*97.4*	*97.6*	93.8	**92.8**	**86.9**
		40	*97.5*	*97.3*	*97.8*	94.2	**92.5**	**88.0**
	400	4	*96.7*	*96.6*	*97.6*	96.2	96.2	**86.1**

**Bolded** values indicate coverage rates below 95%.

*Italicized* values indicate coverage rates above 95%.

Missing data were generated completely at random (MCAR) with a response rate of 60%, and data were imputed using an imputation model that correctly specified the covariate effects.

**Table 2:** Empirical coverage of 95% confidence intervals for the treatment group mean after parametric multiple imputation

ICC	Cluster size	Clusters	NORM	NORM	NORM
*ρ*	*m*	*k*	FE	RE	IGN
0.03	4	100	*99.4*	96.2	95.3
	8	10	*99.6*	*98.0*	*97.4*
		20	*99.2*	96.3	95.6
		40	*99.6*	*96.7*	96.0
	40	10	*99.2*	*97.7*	94.6
		20	*98.5*	95.8	**91.8**
		40	*98.4*	95.1	**91.6**
	400	4	*97.8*	*97.5*	**90.6**
0.08	4	100	*99.3*	95.6	94.8
	8	10	*99.4*	*97.4*	96.0
		20	*98.8*	95.5	93.7
		40	*99.3*	96.1	94.7
	40	10	*98.3*	96.2	**90.9**
		20	*97.6*	94.9	**86.9**
		40	*97.3*	95.2	**88.3**
	400	4	*96.8*	*96.5*	**86.7**

**Bolded** values indicate coverage rates below 95%.

*Italicized* values indicate coverage rates above 95%.

Missing data were generated completely at random (MCAR) with a response rate of 60%, and data were imputed using an imputation model that correctly specified the covariate effects.

#### Model 1b: estimation of regression coefficient with correctly specified mean model

**Fig. 2:**
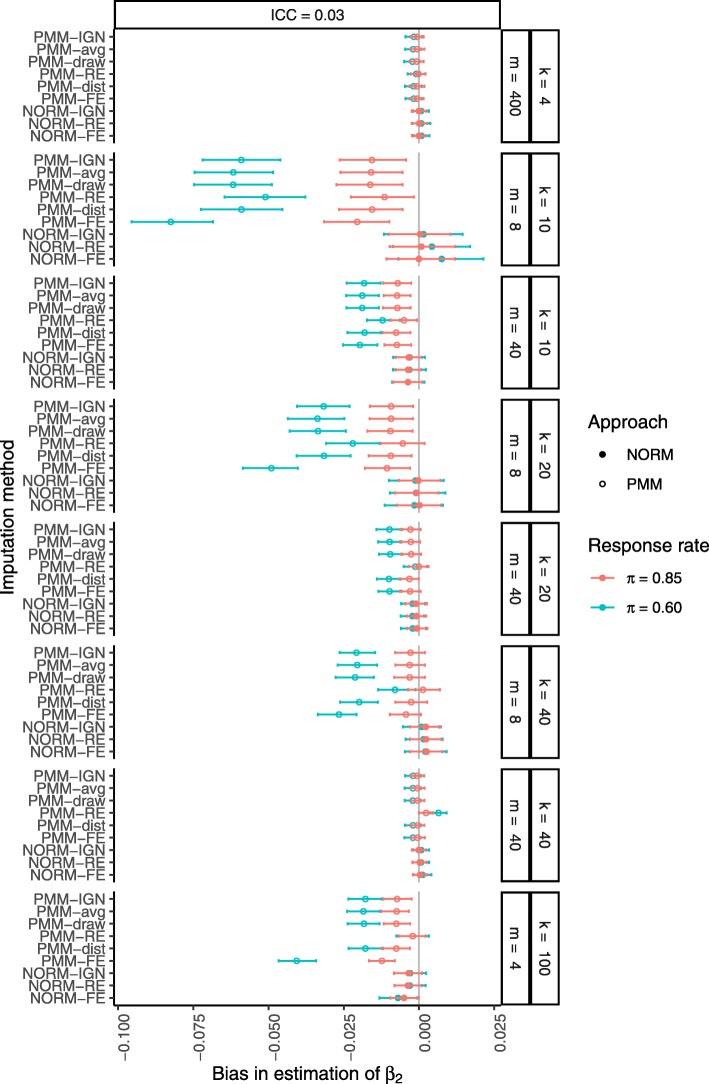
Bias in estimation of *β*_2_ after MI with a correctly specified imputation model. Missing data were generated completely at random (MCAR) at a rate of 15% or 40% (*π*=0.85 in red or *π*=0.60 in green, respectively). From top to bottom, results are presented in increasing order of cluster sizes *m* within an increasing order of number of clusters *k*. Filled circles indicate PMM methods while open circles indicate parametric (NORM) methods

When we moved from estimation of the treatment group mean to estimation of the regression coefficient for the continuous covariate, an unexpected pattern emerged. Figure 2 shows that the parametric procedures produced unbiased estimates of *β*_2_ across all scenarios, but the PMM procedures tended to underestimate the true data-generating value of *β*_2_, which was mitigated by increasing the cluster size or number of clusters. Further investigation revealed that this general underestimation of *β*_2_ with the PMM procedures was complemented by a general *over*estimation of *β*_0_ to the same degree (Supplemental Figure 2), while all methods still produced unbiased estimates of *β*_1_ (not shown). Although this bias was negligible when the rate of missing data was low (within 0.5% of the true value), the magnitude of the bias was two to 10 times larger when the rate of missing data was higher.

As with estimation of the treatment group mean, we saw no meaningful difference in efficiency across methods, this time with the exception of the imputation methods based on fixed effects models. When the rate of missing data was higher, the PMM-FE and NORM-FE procedures yielded relatively higher estimates of the empirical variance (Supplemental Figure 3).

**Fig. 3:**
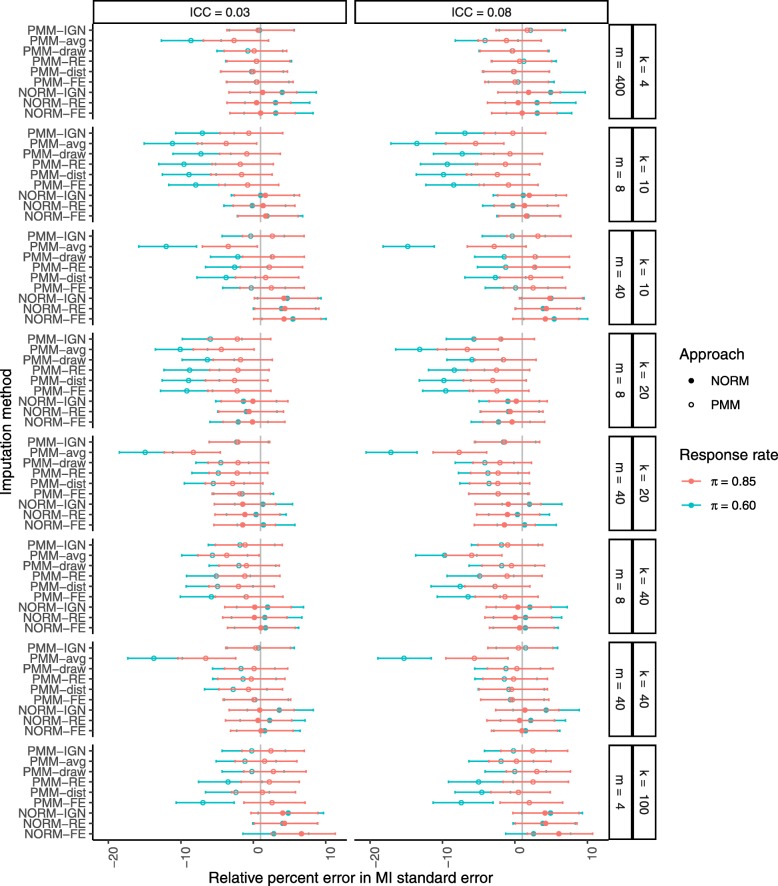
Relative percent error in the MI standard error of *β*_2_ after MI with a correctly specified imputation model. Missing data were generated completely at random (MCAR) at a rate of 15% or 40% (*π*=0.85 in red or *π*=0.60 in green, respectively). From top to bottom, results are presented in increasing order of cluster sizes *m* within an increasing order of number of clusters *k*. Filled circles indicate PMM methods while open circles indicate parametric (NORM) methods

Figure 3 shows the relative percent error in the MI standard error of *β*_2_. Despite the bias in estimation of *β*_2_, all methods except the PMM-avg procedure produced unbiased estimates of its empirical standard error when the rate of missing data was low. The PMM-avg procedure generally underestimated the empirical standard error of *β*_2_, and all other PMM procedures tended to underestimate the empirical standard error when the rate of missing data was higher and the cluster size was smaller (*m*≤8). In contrast, patterns of bias in the standard errors of the other two regression coefficients matched those of the standard error of the treatment group mean (see Supplemental Figure 4), but the magnitudes of the bias in the standard errors of the coefficients were much greater.

Coverage rates for 95% confidence intervals for *β*_2_ under a correctly specified imputation model are shown in Tables 3 and 4. As before, the patterns of under- and overcoverage correspond with the relative percent error in the MI standard error in each scenario.

**Table 3:** Empirical coverage of 95% confidence intervals for *β*_2_ after multiple imputation by PMM

ICC	Cluster size	Clusters	PMM	PMM	PMM	PMM	PMM	PMM
*ρ*	*m*	*k*	FE	dist	RE	draw	avg	IGN
0.03	4	100	**91.5**	94.4	94.0	95.1	94.5	94.7
	8	10	94.5	94.1	94.9	95.1	**93.5**	94.7
		20	**92.6**	93.8	93.7	93.9	**93.2**	94.2
		40	**93.1**	93.8	94.7	94.8	94.0	95.1
	40	10	*97.3*	96.1	*97.0*	*97.0*	94.3	*97.3*
		20	95.8	93.7	94.6	95.1	**91.7**	95.1
		40	95.4	94.8	94.8	94.6	**92.1**	95.3
	400	4	*100*	*100*	*100*	*100*	*99.7*	*100*
0.08	4	100	**91.8**	94.2	**93.2**	95.4	93.8	94.8
	8	10	94.0	94.3	94.5	95.0	**92.5**	94.8
		20	**92.4**	**93.3**	94.3	94.5	**91.9**	94.0
		40	**93.3**	**92.7**	93.8	95	**92.2**	95.0
	40	10	*97.3*	*96.5*	*97*	*96.8*	**93.0**	*97.3*
		20	95.5	94.5	95.5	94.8	**90.4**	95.2
		40	95.2	95.1	94.9	94.8	**91.2**	95.6
	400	4	*100*	*100*	*100*	*100*	*100*	*100*
**Bolded** values indicate coverage rates below 95%.								
*Italicized* values indicate coverage rates above 95%.								
Missing data were generated completely at random (MCAR) with a response rate of 60%, and data were imputed using an imputation model that correctly specified the covariate effects.

**Table 4:** Empirical coverage of 95% confidence intervals for *β*_2_ after parametric multiple imputation

ICC	Cluster size	Clusters	NORM	NORM	NORM
*ρ*	*m*	*k*	FE	RE	IGN
0.03	4	100	95.3	*96.4*	96.1
	8	10	*97.7*	*97.7*	*97.1*
		20	95.8	*97.0*	*97.0*
		40	*96.5*	96.2	*96.4*
	40	10	*98.7*	*98.4*	*98.4*
		20	*96.4*	*96.5*	*96.8*
		40	95.7	95.8	95.9
	400	4	*100*	*100*	*100*
0.08	4	100	95.2	*96.5*	96.2
	8	10	*97.6*	*97.6*	*96.9*
		20	95.9	*96.9*	*96.9*
		40	96.2	95.9	*96.5*
	40	10	*98.6*	*98.3*	*98.4*
		20	*96.4*	*96.6*	*96.7*
		40	95.8	95.6	96.1
	400	4	*100*	*100*	*100*
**Bolded** values indicate coverage rates below 95%.					
*Italicized* values indicate coverage rates above 95%.					
Missing data were generated completely at random (MCAR) with a response rate of 60%, and data were imputed using an imputation model that correctly specified the covariate effects.					

Given the poor performance of the PMM procedures in estimating *β*_2_, we did not further investigate covariate effect estimation under a misspecified imputation model.

#### Model 2: estimation of treatment group mean with misspecified mean model

In estimating the treatment group mean and its variance, the PMM procedures showed small improvements over parametric methods under MCAR when the imputation model was correctly specified. When the imputation model covariate effects were misspecified (missing the squared term in the model), we saw greater disparities among methods.

**Fig. 4:**
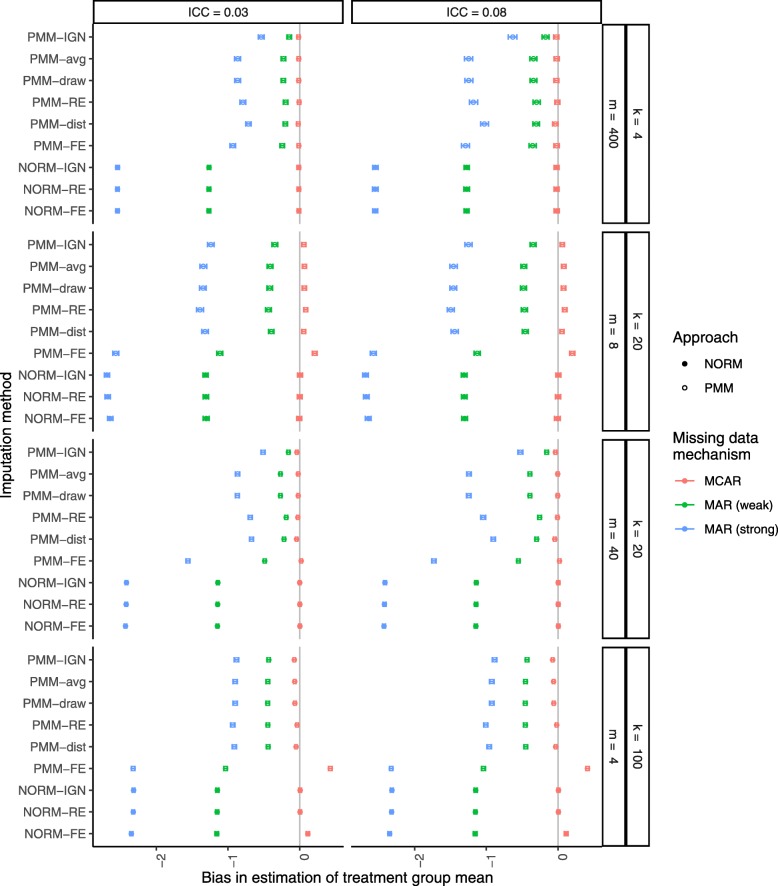
Bias in the treatment group mean estimate after MI with a misspecified imputation model. Missing data were generated completely at random (MCAR in red) or at random (weak MAR in green or strong MAR in blue) at a rate of 40%. From top to bottom, results are presented in increasing order of cluster sizes *m* within an increasing order of number of clusters *k*. Filled circles indicate PMM methods while open circles indicate parametric (NORM) methods

Figure 4 shows the bias in the estimates of the treatment group mean after multiple imputation with a misspecified imputation model. When the data were MCAR, most methods produced little or no bias in the estimates of the treatment group mean, and parametric methods performed better than PMM methods. Under MAR, all methods underestimated the treatment group mean, and this bias was worse under strong MAR. However, the PMM procedures greatly outperformed the parametric procedures under MAR, cutting the bias by more than half in most cases.

Performance of these methods appeared to flip when estimating the empirical variance of the treatment group mean. Under MCAR, the PMM methods were noticeably more efficient than the parametric imputation methods in most cases, but the parametric methods were considerably more efficient under MAR, especially under strong MAR (Supplemental Figure 6).

**Fig. 5:**
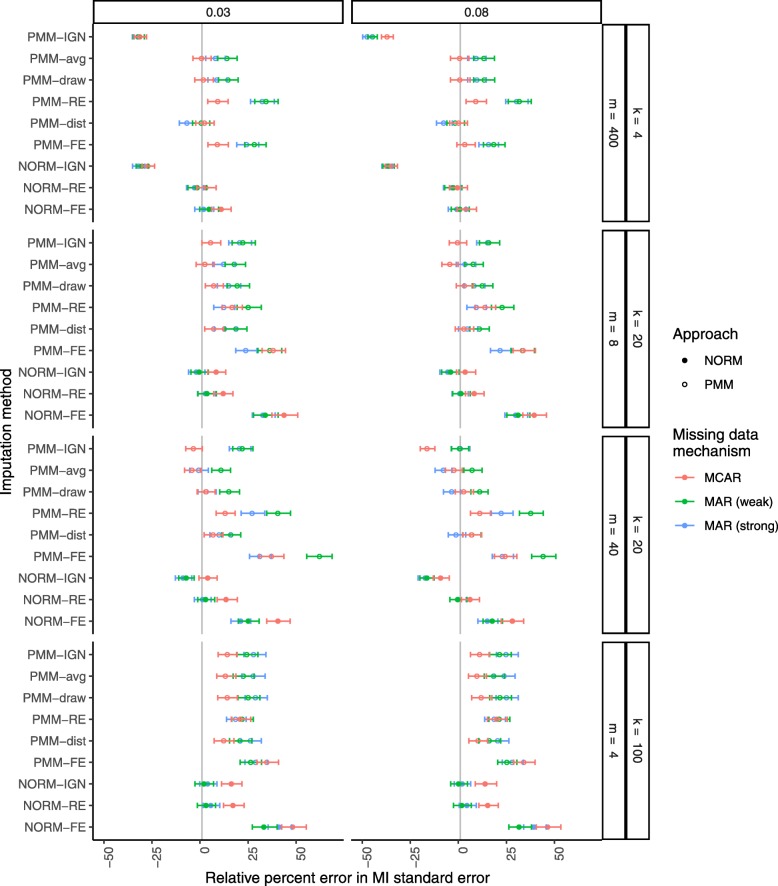
Relative percent error in the MI standard error of the treatment group mean after MI with a misspecified imputation model. Missing data were generated completely at random (MCAR in red) or at random (weak MAR in green or strong MAR in blue) at a rate of 40%. From top to bottom, results are presented in increasing order of cluster sizes *m* within an increasing order of number of clusters *k*. Filled circles indicate PMM methods while open circles indicate parametric (NORM) methods

The relative percent error in the MI standard error is shown in Fig. 5. Under MCAR, the parametric procedures showed greater bias in estimation of the empirical standard error than the PMM procedures. As before, the empirical standard error was generally underestimated for imputation methods that ignored clustering and overestimated for imputation methods that used fixed effects for clusters. Bias in estimation of the empirical standard error was generally worse under weak MAR compared to strong MAR.

Due to the severe underestimation of the treatment group means in most cases, we did not consider coverage of the corresponding 95% confidence intervals.

### Data example: work family and health study

We illustrate our proposed imputation methods using data from a Fortune 500 company that participated in the Work, Family, and Health Study (WFHS; [21]), a multisite cluster-randomized trial designed to evaluate the impact of a workplace intervention on employees’ conflict spillover from work to family or family to work, risk of cardiovascular disease, sleep patterns, and psychological distress. The WFHS included 56 groups of employees ranging in size from 3 to 42 employees per group (median group size of 12 and mean of about 14), where all employees in a group reported to the same leadership. Twenty-seven study groups were assigned to the workplace intervention, while the remaining 29 groups continued business as usual.

We reanalyzed data from a study reporting results after six months of followup, focusing on the measure of control over work hours (CWH) since it had the greatest difference between treatment groups (employees in the intervention reported more control over their work hours) [23]. The study began with 799 participants who completed the baseline CWH survey, but only 694 provided data for the 6-month followup, meaning 13% of the participants were excluded from the available case analysis reported in [23]. The authors modeled baseline and follow-up data with random effects models using random cluster- and subject-specific intercepts as well as random slopes for the time effect [4, 23]. In addition to time (baseline, follow-up), the intervention indicator, and their interaction, the model also included two factors used as part of the randomization scheme: core job function (software developers vs. not) and cluster size. To simplify the analysis and align it with our simulation study, we fit a random effects model predicting CWH at 6 months post-intervention with random cluster-specific intercepts. Baseline CWH, treatment group, core job function, and cluster size were included as covariate effects.

Using the ANOVA method on the available cases, the estimate of the ICC for CWH at 6 months was $\hat \rho =0.14$. Given this moderate-to-large ICC and the moderate cluster sizes, we expect the newly proposed PMM procedures—and the PMM-draw procedure in particular—to produce fairly consistent estimates across analyses, and these variance estimates should be closer to the truth than those produced from the established imputation procedures.

We first fit our model with an available case analysis to coincide with the approach described in [23]. Though not directly comparable due to the difference in models, results from the available case analysis under our fitted model were similar to the results from the model fit in the original analysis in terms of direction and significance of effects [23]. Specifically, neither core job function nor cluster size were significant at the 0.05 level, but there was a significant effect of the workplace intervention ($\hat \beta = 0.19, p = < 0.001$; Table 5).

**Table 5:** Available case analysis of the Work, Family, and Health Study (WFHS) data

Effect	Estimate	SE	df	*t* value	P-value
Intercept	1.2708	0.1166	300.27	10.8946	<0.001
Intervention	0.1932	0.0492	43.83	3.9255	<0.001
Baseline CWH	0.6581	0.0283	655.70	23.2595	<0.001
Core function	0.0265	0.0491	39.99	0.5395	0.5926
Cluster size	-0.0001	0.0020	35.85	-0.0391	0.969

We compared these results to analyses after multiple imputation of the data using the nine MI methods considered in our simulation study. Twenty imputations were used for each method. The imputation models included all covariate effects that were used in the analysis model, five additional covariates that were associated with CWH (job authority, time adequacy, work-to-family conflict spillover, supervisor supportive behaviors, and burnout), and two variables associated with the likelihood of missing CWH at six months (job demands and psychological distress). Consistent with the results of our simulation study, estimates of the intervention effect were similar for all imputation methods (range: 0.188−0.206; Table 6). As expected based on our analytical results, the standard errors after multiple imputation were smallest in methods that ignored clustering and largest in fixed effects methods. The standard errors were also comparable among the three newly proposed procedures, with estimates of 0.048, 0.049, and 0.050 for the PMM-avg, PMM-draw, and PMM-dist procedure, respectively. Importantly, our conclusion about the intervention effect remained the same across all imputation methods: the WFHS intervention significantly increased perceived control over work hours, with a p-value <.001 (Table 6).

**Table 6:** Results after correctly-specified multiple imputation of the CWH outcome in the WFHS

Method	Intervention effect	Pooled SE	P-value
PMM-IGN	0.1962	0.0473	<0.001
PMM-RE	0.1883	0.0526	<0.001
PMM-avg	0.1990	0.0475	<0.001
PMM-draw	0.2012	0.0490	<0.001
PMM-dist	0.1920	0.0504	<0.001
PMM-FE	0.2058	0.0553	<0.001
NORM-IGN	0.1907	0.0456	<0.001
NORM-RE	0.2011	0.0507	<0.001
NORM-FE	0.2041	0.0580	<0.001

As a further check of robustness, we analyzed the data again with only the intervention indicator as a covariate effect in the imputation models (see Table 7). In this case, the estimates of the intervention effect all decreased while the standard errors after multiple imputation all increased. The standard errors after multiple imputation remained smallest in methods that ignored clustering and largest in methods that used fixed effects for clusters, and there were smaller disparities between the three newly proposed procedures relative to the established methods. Again, these results are consistent with the biases we saw in the second part of our simulation study.

**Table 7:** Results after misspecified multiple imputation of the CWH outcome in the WFHS

Method	Intervention effect	Pooled SE	P-value
PMM-IGN	0.1760	0.0524	<0.001
PMM-RE	0.1834	0.0619	0.0032
PMM-avg	0.1767	0.0533	0.001
PMM-draw	0.1855	0.0570	0.0012
PMM-dist	0.1856	0.0613	0.0026
PMM-FE	0.1852	0.0640	0.0039
NORM-IGN	0.1795	0.0523	<0.001
NORM-RE	0.1825	0.0598	0.0024
NORM-FE	0.1906	0.0626	0.0024

## Discussion

Our goal was to develop a new PMM procedure that could handle missing data from a two-level cluster-randomized trial and would be robust to misspecification of the imputation model. We showed that, relative to the PMM-RE procedure and most other imputation methods that have been used for multilevel multiple imputation, our three newly proposed procedures generally improved estimation of the variance of the treatment group mean when the data were missing completely at random. Parametric procedures actually produced better estimates of the variance of the treatment group mean when the data were missing at random and the imputation model was misspecified, but did so at the cost of greater bias in estimation of the treatment group mean relative to the predictive mean matching procedures.

Among the newly proposed PMM procedures, we are particularly fond of the PMM-draw method. It outperformed all other methods when the imputation model was correctly specified, producing unbiased estimates of the treatment group mean and its variance and maintaining nominal coverage of 95% confidence intervals of the treatment group mean. Keeping in mind the bias-variance tradeoff issues mentioned earlier, the three newly proposed procedures outperformed all other procedures when the imputation model was misspecified. Although the three procedures performed similarly in this setting, the PMM-draw procedure is still recommended over the other two since it can be readily implemented in available software and, like traditional PMM procedures, only imputes plausible values.

Performance of the PMM procedures in estimating regression coefficients in the linear model varied widely depending on the coefficient and the rate of missing data. Although the bias in the estimates of the intercept and continuous covariate effect was minimal for the PMM procedures, we would recommend using the parametric NORM-RE procedure in particular when estimating regression coefficients from a linear model.

This work was limited to estimation of treatment group means and linear regression coefficients when only the outcome was missing and all covariates were fully observed. Future work should investigate these procedures when covariates are not fully observed or when multiple variables are subject to missing data. It is also important to evaluate these procedures in other settings, such as estimation of logistic regression coefficients. Another limitation of this work is the focus on Normally distributed errors when the Normality assumption is often violated in real applications. He and Raghunathan [24] investigated the performance of multiple imputation procedures that assumed Normality in the presence of skewed data, including parametric imputation and predictive mean matching procedures. They showed that the MI procedures performed well when estimating the mean but could perform poorly when estimating regression coefficients if the underlying distribution was strongly skewed. We expect similar results to hold in the context of cluster randomized trials and with our newly proposed PMM procedures, but future work should investigate these procedures in this setting.

## Conclusion

While our new methods offer an improvement over established methods in estimating the variance, there is still some bias in the variance estimation when data are missing at random and the predictive mean model is misspecified. When estimating the weights for these methods, we used naïve estimates of the bias in the variance that assumed there were no covariates in the data set and that the data were missing completely at random. Additionally, our weights were derived assuming a balanced design (equal number of clusters in each treatment group, and equal number of subjects in each cluster). If an improved estimator can be derived to allow for more relaxed assumptions, we may see further improvement in multiply imputed parameter estimates from these methods.

## Supplementary information

**Additional file 1** The appendix contains the derivation of the formula for the bias in the variance of the treatment group mean after multiple imputation of missing CRT data using an imputation model that ignores clustering.

**Additional file 2** The supplement contains additional figures and tables that were not necessary for the manuscript but may be useful references for the reader.

## Data Availability

The datasets generated and analyzed for this study are available from the corresponding author upon request.

## Abbreviations

CRT: cluster-randomized trial
distributions; FE: fixed effects for clusters
ICC: intracluster correlation coefficient
IGN: ignoring clusters
MAR: missing at random
MCAR: missing completely at random
MI: multiple imputation
MNAR: missing not at random
NORM: indicates a parametric multiple imputation procedure that relies on the assumption of Normality of the error PMM-avg: predictive mean matching via a weighted average of results from the PMM-FE and PMM-IGN procedures
PMM-dist: predictive mean matching with a weighted distance metric
PMM-draw: predictive mean matching via a weighted draw of results from the PMM-FE and PMM-IGN procedures
PMM: predictive mean matching
RE: random effects for clusters
SE: standard error
WFHS: Work, Family, and Health Study
