# Supplementary material for: Multiple imputation by predictive mean matching in cluster-randomized trials
Source: BMC Med Res Methodol. 2020 Mar 30;20:72. doi: 10.1186/s12874-020-00948-6 (PMC7106802; doi:10.1186/s12874-020-00948-6)
Supplement: Supplementary file 1 — Additional file 1 The appendix contains the derivation of the formula for the bias in the variance of the treatment group mean after multiple imputation of missing CRT data using an imputation model that ignores clustering. [file 12874_2020_948_MOESM1_ESM.pdf]

Supplemental material

Supplemental figures

Model 1a: Estimation of treatment group mean with correctly specified mean model

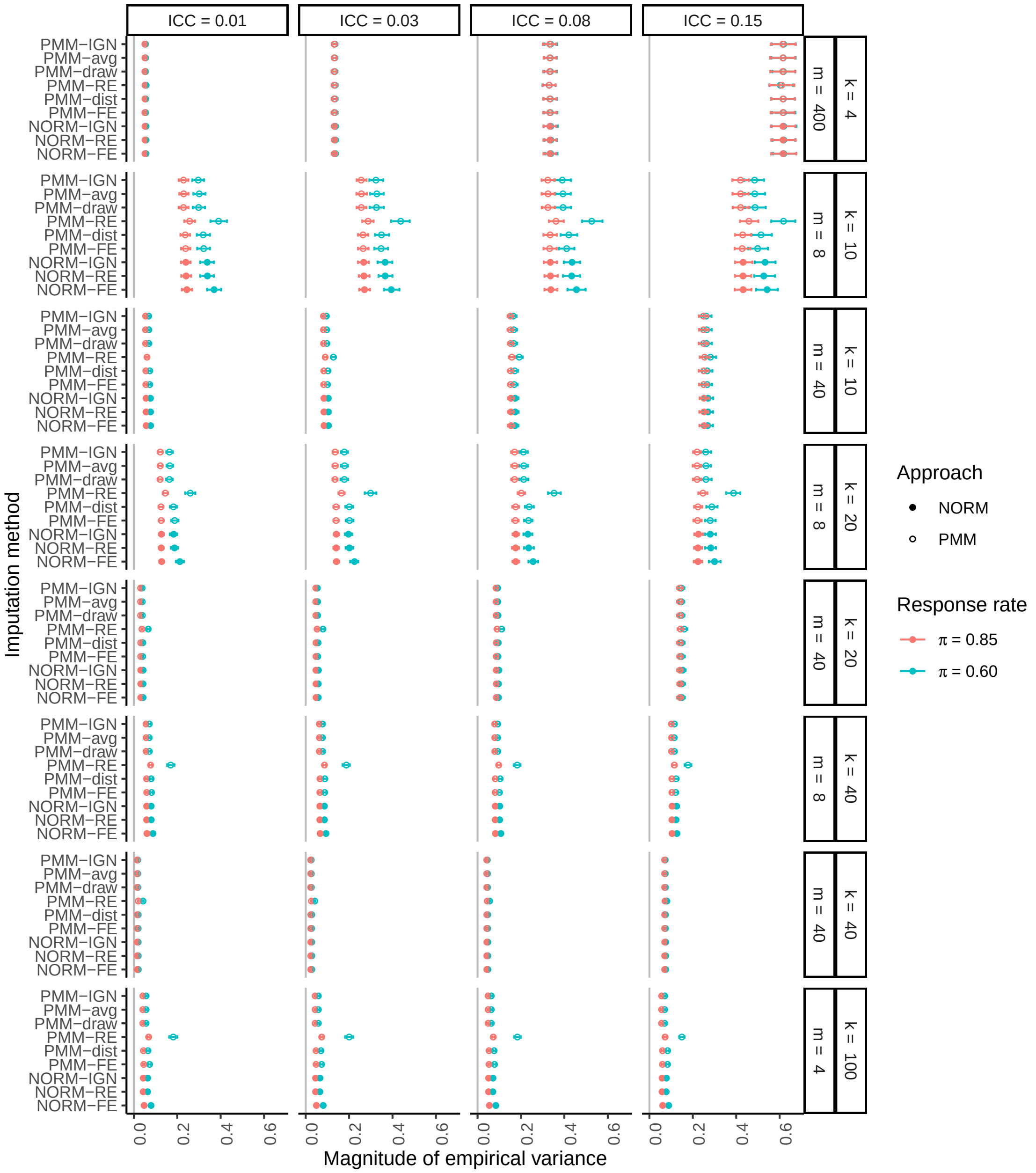

Figure 1 Magnitude of the empirical variance of the treatment group mean after MI with a correctly specified imputation model. Missing data were generated completely at random (MCAR) at a rate of 15% or 40% ($\pi = 0.85$ in red or $\pi = 0.60$ in green, respectively). From top to bottom, results are presented in increasing order of cluster sizes m within an increasing order of number of clusters k. From left to right, results are presented in increasing order of the intracluster correlation coefficient (ICC, ρ). Filled circles indicate PMM methods while open circles indicate parametric (NORM) methods.

Model 1b: Estimation of regression coefficient with correctly specified mean model

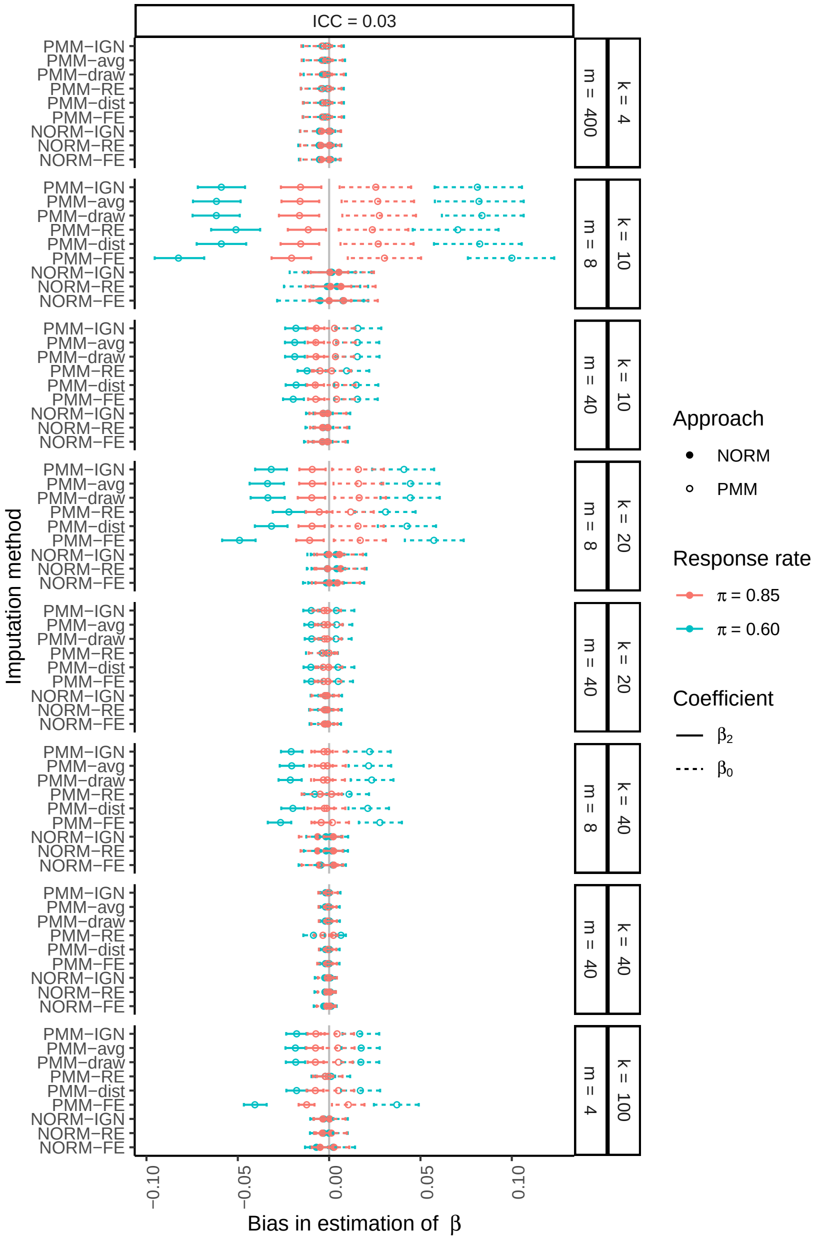

Figure 2 Comparison of bias in estimation of β_2 (solid line) and β_0 (dashed line) after MI with a correctly specified imputation model. Missing data were generated completely at random (MCAR) at a rate of 15% or 40% ($\pi = 0.85$ in red or $\pi = 0.60$ in green, respectively). From top to bottom, results are presented in increasing order of cluster sizes m within an increasing order of number of clusters k. Filled circles indicate PMM methods while open circles indicate parametric (NORM) methods.

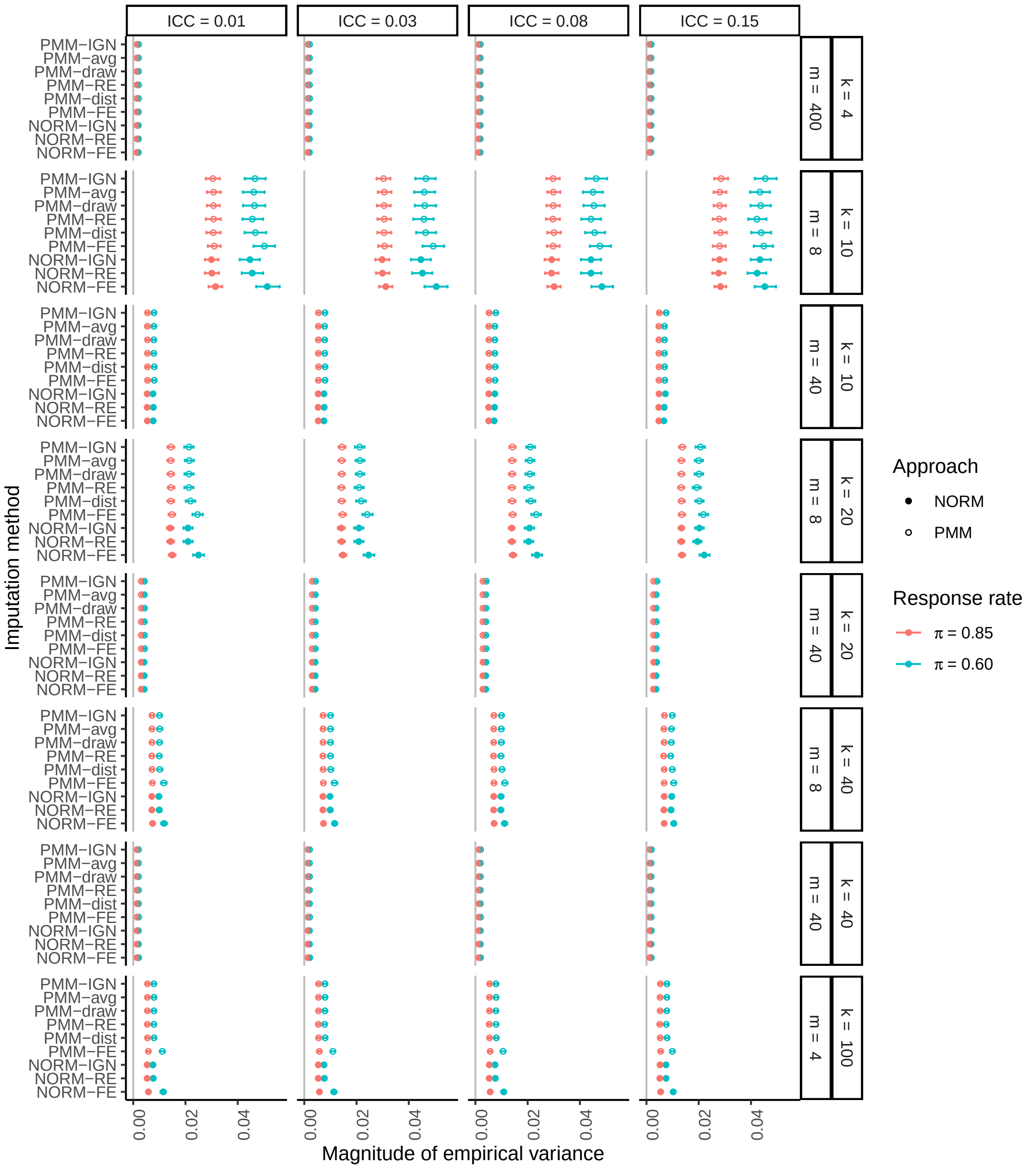

Figure 3 Magnitude of the empirical variance of β_2 after MI with a correctly specified imputation model. Missing data were generated completely at random (MCAR) at a rate of 15% or 40% ($\pi = 0.85$ in red or $\pi = 0.60$ in green, respectively). From top to bottom, results are presented in increasing order of cluster sizes m within an increasing order of number of clusters k. From left to right, results are presented in increasing order of the intracluster correlation coefficient (ICC, ρ). Filled circles indicate PMM methods while open circles indicate parametric (NORM) methods.

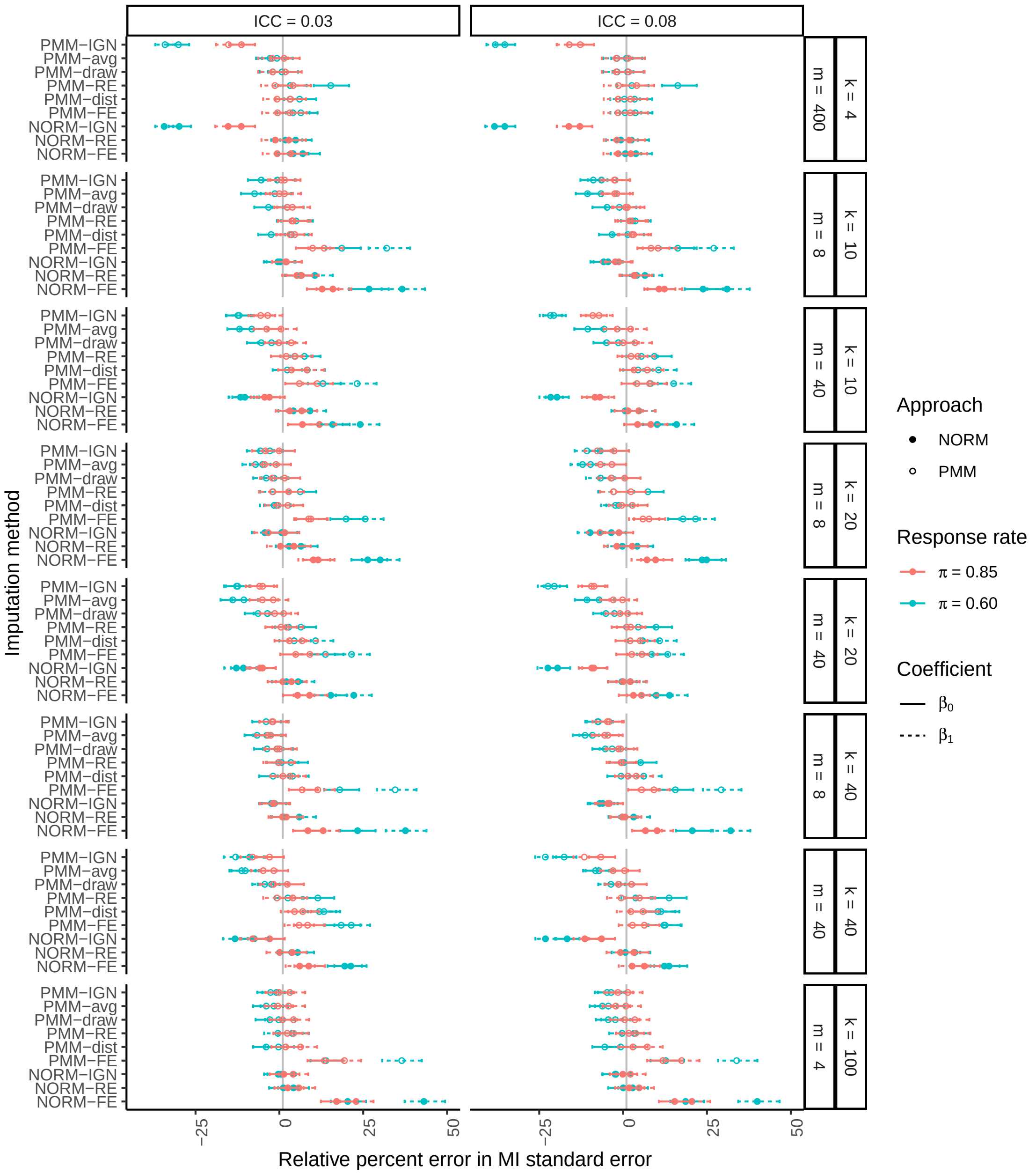

Figure 4 Comparison of relative percent error in the MI standard error of β_0 (solid line) and β_1 (dashed line) after MI with a correctly specified imputation model. Missing data were generated completely at random (MCAR) at a rate of 15% or 40% ($\pi = 0.85$ in red or $\pi = 0.60$ in green, respectively). From top to bottom, results are presented in increasing order of cluster sizes m within an increasing order of number of clusters k. Filled circles indicate PMM methods while open circles indicate parametric (NORM) methods.

Model 2: Estimation of treatment group mean with misspecified mean model

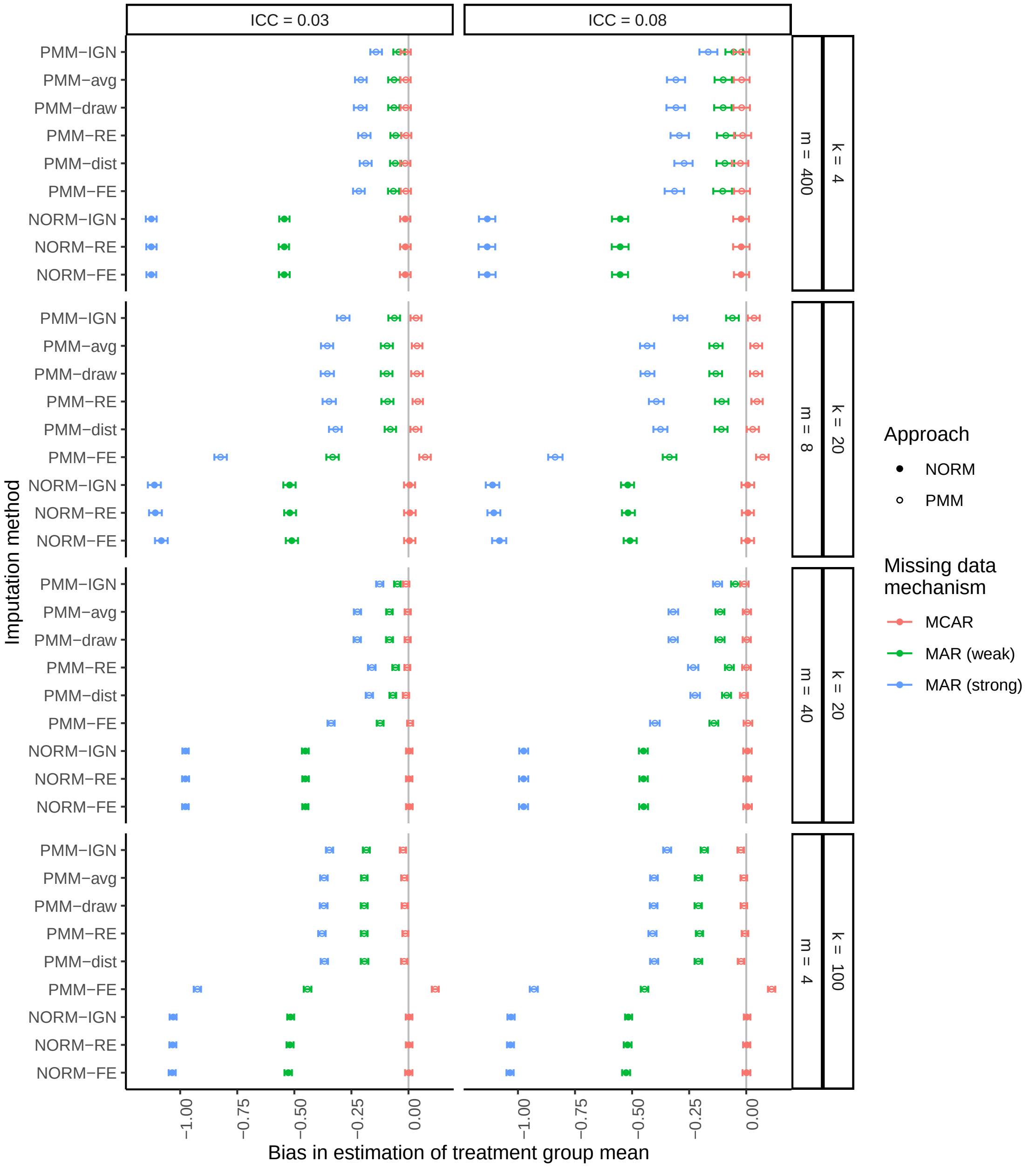

Figure 5 Bias in the treatment group mean estimate after MI with a misspecified imputation model. Missing data were generated completely at random (MCAR in red) or at random (weak MAR in green or strong MAR in blue) at a rate of 15%. From top to bottom, results are presented in increasing order of cluster sizes m within an increasing order of number of clusters k. Filled circles indicate PMM methods while open circles indicate parametric (NORM) methods.

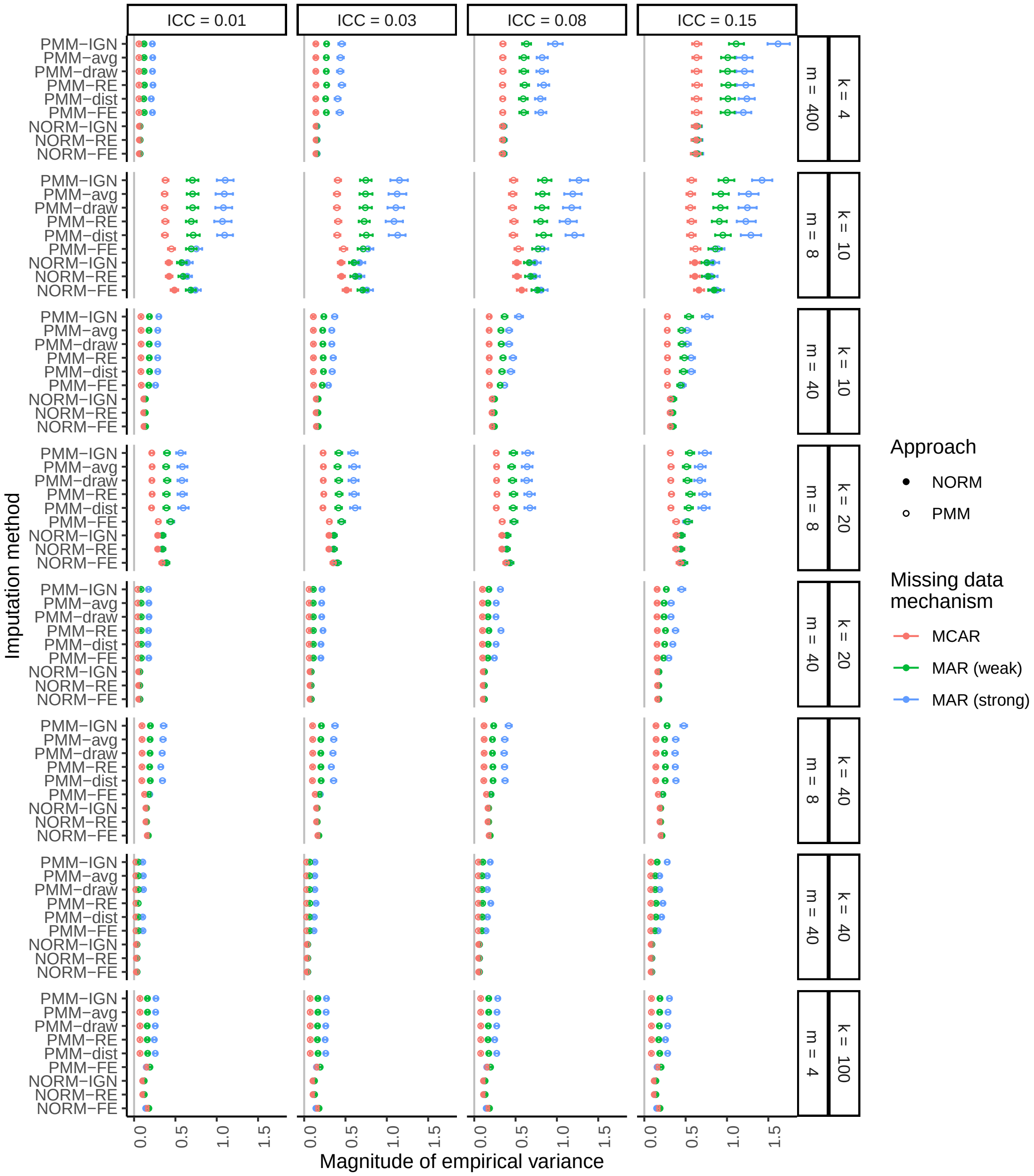

Figure 6 Magnitude of the empirical variance of the treatment group mean after MI with a misspecified imputation model. Missing data were generated completely at random (MCAR in red) or at random (weak MAR in green or strong MAR in blue) at a rate of 40%. From top to bottom, results are presented in increasing order of cluster sizes m within an increasing order of number of clusters k. From left to right, results are presented in increasing order of the intracluster correlation coefficient (ICC, ρ). Filled circles indicate PMM methods while open circles indicate parametric (NORM) methods.

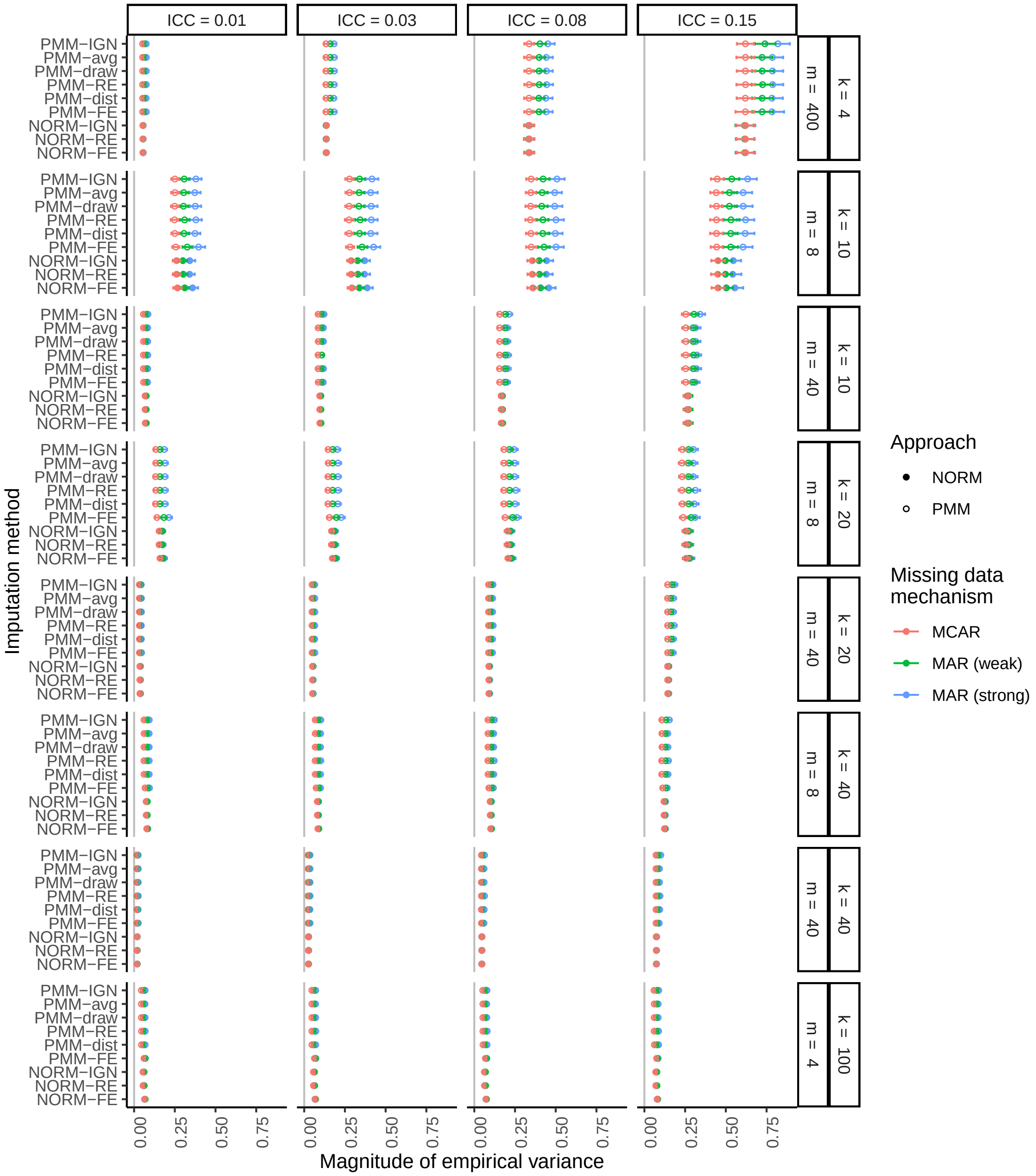

Figure 7 Magnitude of the empirical variance of the treatment group mean after MI with a misspecified imputation model. Missing data were generated completely at random (MCAR in red) or at random (weak MAR in green or strong MAR in blue) at a rate of 15%. From top to bottom, results are presented in increasing order of cluster sizes m within an increasing order of number of clusters k. From left to right, results are presented in increasing order of the intracluster correlation coefficient (ICC, ρ). Filled circles indicate PMM methods while open circles indicate parametric (NORM) methods.

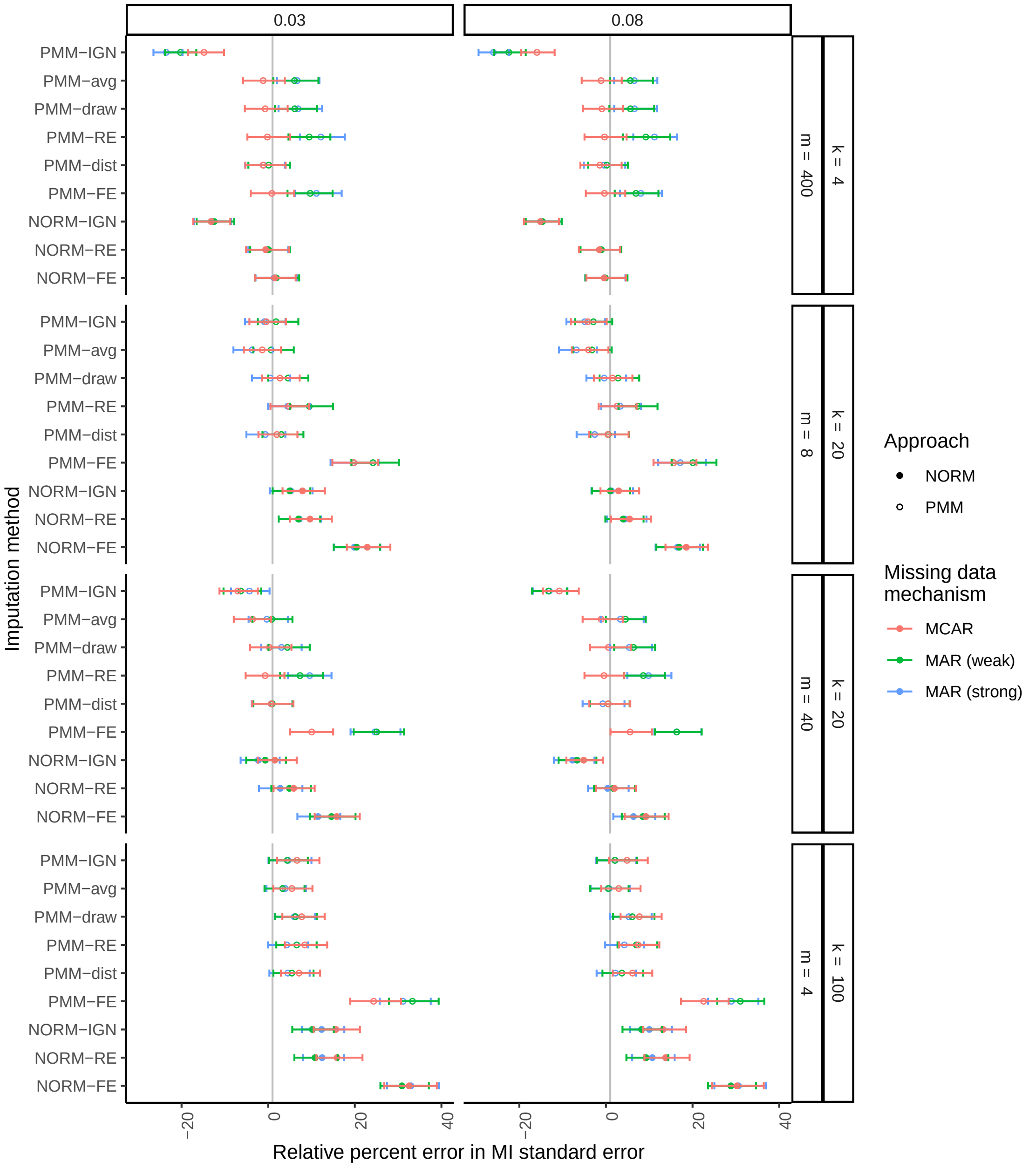

Figure 8 Relative percent error in the MI standard error of the treatment group mean after MI with a misspecified imputation model. Missing data were generated completely at random (MCAR in red) or at random (weak MAR in green or strong MAR in blue) at a rate of 15%. From top to bottom, results are presented in increasing order of cluster sizes m within an increasing order of number of clusters k. Filled circles indicate PMM methods while open circles indicate parametric (NORM) methods.

Supplemental tables

Model 1a: Estimation of treatment group mean with correctly specified mean model

Table 1 Empirical coverage of 95% confidence intervals for the treatment group mean after multiple imputation by PMM.

ICC rho	Cluster size m	No. clusters k	PMM FE	PMM dist	PMM RE	PMM draw	PMM avg	PMM IGN
0.03	4	100	*98.4*	*98.1*	*96.6*	*96.9*	96.3	*96.4*
0.03	8	10	*98*	*97.6*	*97.5*	*97.3*	*97.1*	*97*
0.03	8	20	*97.1*	95.9	95.2	95.4	94.2	94.9
0.03	8	40	*97.1*	*96.8*	95.7	96	95.4	95.5
0.03	40	10	*97.9*	*97.7*	*97.2*	*96.8*	*96.4*	96.1
0.03	40	20	95.9	95.9	95.4	94.6	93.9	93.8
0.03	40	40	*97.1*	*96.8*	96	96	95.4	94.7
0.03	400	4	96.2	96.3	*96.4*	96	96	94.6
0.08	4	100	*98.5*	*98.3*	*97.1*	*96.6*	95.8	96.1
0.08	8	10	*97.4*	*97.2*	*97.2*	*96.6*	96.2	95.7
0.08	8	20	*96.6*	96.2	95	94.1	**93.4**	**93.6**
0.08	8	40	*96.9*	*96.7*	95.8	95.4	94.8	95
0.08	40	10	*97.2*	*97.1*	*97*	*96.8*	*96.4*	94.5
0.08	40	20	95.8	95.6	95.7	94.9	94.4	**93**
0.08	40	40	*96.4*	96.2	95.9	95.5	95.5	**93.6**
0.08	400	4	*96.4*	*96.5*	*96.5*	96.2	96.3	93.9

Bolded values indicate coverage rates below 95%.
Italicized values indicate coverage rates above 95%.
Missing data were generated completely at random with a response rate of 85%, and data were imputed using an imputation model that correctly specified the covariate effects.

Table 2 Empirical coverage of 95% confidence intervals for the treatment group mean after parametric multiple imputation.

ICC ρ	Cluster size m	No. clusters k	NORM FE	NORM RE	NORM IGN
0.03	4	100	*98.6*	*97*	96.3
0.03	8	10	*98.5*	*97.2*	*97.6*
0.03	8	20	*97.7*	95.1	94.5
0.03	8	40	*97.8*	95.9	95.3
0.03	40	10	*97.8*	*97.1*	95.6
0.03	40	20	96	94.9	93.9
0.03	40	40	*97.3*	96	94.3
0.03	400	4	96.3	96.1	94.5
0.08	4	100	*98.5*	*96.9*	95.8
0.08	8	10	*98.1*	*96.8*	96
0.08	8	20	*97.1*	94.5	93.9
0.08	8	40	*97.1*	95.5	94.7
0.08	40	10	*97.1*	*97*	94.5
0.08	40	20	95.8	94.9	**93**
0.08	40	40	96.3	95.7	**93.4**
0.08	400	4	*96.5*	*96.4*	94

Bolded values indicate coverage rates below 95%.
Italicized values indicate coverage rates above 95%.
Missing data were generated completely at random with a response rate of 85%, and data were imputed using an imputation model that correctly specified the covariate effects.

Model 1b: Estimation of regression coefficient with correctly specified mean model

Table 3 Empirical coverage of 95% confidence intervals for β_2 after multiple imputation by PMM.

ICC rho	Cluster size m	No. clusters k	PMM FE	PMM dist	PMM RE	PMM draw	PMM avg	PMM IGN
0.03	4	100	95.6	95.3	*96.8*	96.1	95.6	96.1
0.03	8	10	*97.5*	*97*	*96.5*	*97.3*	*96.5*	*97.2*
0.03	8	20	95.1	94.1	94.5	94.9	93.8	94.6
0.03	8	40	94.7	94.5	94.8	95.1	94.5	95
0.03	40	10	*97.7*	*97.5*	*97.6*	*97.9*	*96.8*	*97.8*
0.03	40	20	96.2	96.1	96.3	*96.5*	94.1	96
0.03	40	40	95.6	95.2	95.4	95.6	93.9	96.3
0.03	400	4	*99.6*	*99.6*	*99.6*	*99.6*	*99.3*	*99.6*
0.08	4	100	96	95.5	*96.4*	*96.4*	95.3	95.9
0.08	8	10	*97.2*	*96.7*	*97.1*	*97.6*	95.9	*97*
0.08	8	20	94.9	94.2	94.5	95	**93.3**	94.5
0.08	8	40	94.5	94.5	94.7	95	93.9	95.3
0.08	40	10	*97.7*	*97.7*	*97.7*	*97.9*	*97.2*	*97.7*
0.08	40	20	96.3	96.1	96.1	96.1	94.5	96.1
0.08	40	40	95.3	95.3	95.5	95.6	93.8	95.7
0.08	400	4	*99.7*	*99.8*	*99.8*	*99.8*	*99.7*	*99.7*

Bolded values indicate coverage rates below 95%.

Italicized values indicate coverage rates above 95%.

Missing data were generated completely at random (MCAR) with a response rate of 85%, and data were imputed using an imputation model that correctly specified the covariate effects.

Table 4 Empirical coverage of 95% confidence intervals for β_2 after parametric multiple imputation.

ICC<br>ρ	Cluster size<br>m	No. clusters<br>k	NORM<br>FE	NORM<br>RE	NORM<br>IGN
0.03	4	100	*97.3*	*96.6*	95.9
0.03	8	10	*97.6*	*97.6*	*97.3*
0.03	8	20	95.3	95.2	95.3
0.03	8	40	95.7	95.6	95.6
0.03	40	10	*98*	*98*	*97.9*
0.03	40	20	*96.4*	*96.5*	96.3
0.03	40	40	95.8	95.5	96.1
0.03	400	4	*99.8*	*99.9*	*99.8*
0.08	4	100	*97.1*	*96.6*	96.1
0.08	8	10	*97.5*	*97.5*	*97.4*
0.08	8	20	95.3	95.2	95.4
0.08	8	40	95.7	95.8	95.6
0.08	40	10	*98*	*97.9*	*98*
0.08	40	20	96.3	*96.5*	*96.6*
0.08	40	40	95.7	95.3	96.3
0.08	400	4	*99.8*	*99.9*	*99.8*

Bolded values indicate coverage rates below 95%.
Italicized values indicate coverage rates above 95%.
Missing data were generated completely at random (MCAR) with a response rate of 85%, and data were imputed using an imputation model that correctly specified the covariate effects.
